# Clinical study of melodic intonation therapy combined with transcranial direct current stimulation for post-stroke aphasia: a single-blind, randomized controlled trial

**DOI:** 10.3389/fnins.2023.1088218

**Published:** 2023-06-15

**Authors:** Zhijie Yan, Xinyuan He, Mangmang Cheng, Xiaoqing Fan, Dongshuai Wei, Shuo Xu, Chong Li, Xiaofang Li, Hongxia Xing, Jie Jia

**Affiliations:** ^1^Department of Rehabilitation Medicine, The Third Affiliated Hospital of Xinxiang Medical University, Xinxiang, China; ^2^Department of Rehabilitation Medicine, Huashan Hospital, Fudan University, Shanghai, China; ^3^National Clinical Research Center for Aging and Medicine, Huashan Hospital, Fudan University, Shanghai, China

**Keywords:** stroke, aphasia, tDCS, melodic intonation therapy, closed-loop rehabilitation

## Abstract

**Background:**

Globally, more than 10 million new stroke cases occur annually, of which aphasia accounts for about one-third. Aphasia has become an independent predictor of functional dependence and death for the stroke population. The closed-loop rehabilitation of combining behavioral therapy with central nerve stimulation seems to be the research trend of post-stroke aphasia (PSA) due to its advantages in improving linguistic deficits.

**Objective:**

To verify the clinical efficacy of a closed-loop rehabilitation program combining melodic intonation therapy (MIT) with transcranial direct current stimulation (tDCS) for PSA.

**Methods:**

This was a single-center, assessor-blinded, randomized controlled clinical trial, which screened 179 patients and included 39 PSA subjects, with the registration number ChiCTR2200056393 in China. Demographic and clinical data were documented. The primary outcome was the Western Aphasia Battery (WAB) used to assess language function, and the secondary outcomes included Montreal Cognitive Assessment (MoCA), Fugl-Meyer Assessment (FMA), and Barthel Index (BI) for evaluating cognition, motor, and activities of daily living, respectively. With the computer-generated randomization sequence, subjects were randomly divided into the conventional group (CG), MIT combined with sham stimulation group (SG), and MIT combined with tDCS group (TG). After the three-week intervention, the functional changes in each group were analyzed by the paired sample *T*-test, and the functional difference between the three groups was analyzed by ANOVA.

**Results:**

There was no statistical difference on the baseline. After the intervention, the WAB’s aphasia quotient (WAB-AQ), MoCA, FMA, and BI were statistically different in SG and TG, including all the sub-items in WAB and FMA, while only listening comprehension, FMA, and BI were statistically different in CG. The differences of WAB-AQ, MoCA, and FMA were statistically different among the three groups, but BI was not. The *post hoc* test results revealed that the changes of WAB-AQ and MoCA in TG were more significant than the others.

**Conclusion:**

MIT combined with tDCS can augment the positive effect on language and cognitive recovery in PSA.

## 1. Introduction

Globally, more than 10 million new stroke cases occur annually, of which post-stroke aphasia (PSA) accounts for about one-third (Stefaniak et al., 2020). About 21–40% of stroke patients have persistent language impairment symptoms, which seriously affect the quality of life (Cichon et al., 2021). Research has shown that aphasia is an independent predictor of functional dependence and death after stroke, which has led to aphasia, an acquired language defect, becoming a clinical problem to be solved urgently (Stefaniak et al., 2020). At present, speech-language therapy (SLT) in behavioral science is still the mainstream treatment for aphasia (Fridriksson and Hillis, 2021). High-dose and high-intensity SLT is beneficial to the functional recovery of PSA (Breitenstein et al., 2017), but when SLT is more than 2 h per day, the benefit does not increase significantly (Rehabilitation and Recovery of People with Aphasia after Stroke, 2022). In the context of limited medical resources, treatment strategies need to be reconsidered based on the existing evidence to maximize language recovery and cost-effectiveness.

The closed-loop rehabilitation is a treatment paradigm designed based on the central-peripheral-central concept, which organically combines central nervous system regulation with peripheral therapy to compensate for the limitations of simple peripheral or central intervention. In closed-loop rehabilitation that can activate functional pathways, the central intervention represented by non-invasive brain stimulation (NIBS) can promote the activation of corresponding brain functional areas and enhance neuroplasticity, and peripheral intervention represented by behavioral therapy strengthens positive feedback through continuous input of motor and sensory signals, and some scholars believe that neuroimmunology mechanisms may also be involved (Jia, 2022). Several studies provide clues to closed-loop treatment strategies that boost functional recovery in aphasia (Vines et al., 2011; Wang et al., 2014; Fridriksson et al., 2018a,b; Cichon et al., 2021; Sheppard and Sebastian, 2021). However, the combination of peripheral and central interventions requires more evidence of randomized controlled trials to support its clinical implementation.

Multi-center cross-sectional study shows that there is a positive correlation between upper limb motor function and language function in the stroke population (Xu et al., 2021), which may suggest giving priority to treatment models involving both upper limb movement and language. Melodic Intonation Therapy (MIT) can be applied to aphasia, especially non-fluent aphasia caused by ischemic damage in the left dominant hemisphere (American Academy of Neurology, 1994; Cichon et al., 2021). MIT mainly emphasizes the use of external features of speech, such as intonation, rhythm, stress, etc., to recite polysyllabic words, high-frequency phrases, long sentences, and complex sentences musically, and finally to communicate in a normal rhythm and intonation (Cichon et al., 2021). During the treatment, allow the patient to clap according to the rhythm (Haro-Martinez et al., 2019). Compared with conventional speech therapy, MIT, as a behavioral therapy, has shown its advantages in the functional recovery of spontaneous, comprehension, repetition, naming, communication, and other aspects (Kasdan and Kiran, 2018; Huang et al., 2021; Zhang X. Y. et al., 2021; Zhang X. et al., 2021; Siponkoski et al., 2023). With the onset of PSA, the earlier MIT gets involved, the more significant this advantage will be (van der Meulen et al., 2014, 2016). The researches show that MIT is an intervention that is expected to improve language ability in the long run and is effective for many language families (Cortese et al., 2015; Martzoukou et al., 2021; Zhang X. Y. et al., 2021; Zhang X. et al., 2021).

In addition, NIBS has become an important auxiliary means for the treatment of PSA. There is evidence that NIBS is effective for aphasia symptoms and augments the effect of behavioral therapy through a neural plasticity mechanism (Fridriksson and Hillis, 2021). Transcranial direct current stimulation (tDCS), as a technology in NIBS, has great potential in central regulation (Duncan et al., 2020). The results of the systematic review and meta-analysis show that the most effective intervention of tDCS in improving language function may be to stimulate the inferior frontal gyrus (IFG) of the left dominant hemisphere with the anode (Elsner et al., 2020). Its mechanism is that the tDCS anode depolarizes the neuron cell membrane, thereby enhancing cortical activity (Duncan et al., 2020; Cichon et al., 2021). It is generally believed that tDCS is a therapeutic method with minimal risk and may be one of the most promising therapeutic methods besides language training (Bikson et al., 2016).

To optimize the outcome of rehabilitation, this trial was designed to verify whether tDCS anode stimulation of left IFG combined with MIT can augment the positive effect and provide a more evidence-based basis for aphasia rehabilitation.

## 2. Materials and methods

### 2.1. Trial design and sample size calculation

This was a single-center, assessor-blinded, three-arm, randomized controlled trial, granted by the Ethics Committee of the Third Affiliated Hospital of Xinxiang Medical University (ethical approval no. K2021-040-01), with the registration number ChiCTR220056393 in China. The software of Power Analysis and Sample Size Version 15.0.5 (PASS V15.0.5) was applied to calculate the sample size. The enrolled participants were evenly allocated to three arms in a ratio of 1:1:1. Based on the previous research results, the estimated effect size was 0.58. With the 0.05 two-sided significance level (*α* = 0.05) and an 80% power of the test (*β* = 0.2), 13 cases per arm were calculated considering a 15% dropout rate.

### 2.2. Participants

39 PSA from the Third Affiliated Hospital of Xinxiang Medical University were recruited between December 2021 and October 2022.

The inclusion criteria were as follows: (a) right-handedness, (b) Chinese as the first language, (c) first stroke, (d) ischemic lesion of the left hemisphere, (e) course >1 week, (f) aphasia quotient (AQ) of Western Aphasia Battery (WAB) lower than 93.8, (g) non-fluent aphasia demonstrated by the Chinese aphasia fluency characteristics scale score of 9–13, (h) can cooperate to complete all assessments and treatments, and sign informed consent.

The exclusion criteria were as follows: (a) recurrent stroke, (b) history of brain surgery or metal implantation, (c) history of seizures, (d) sensitive scalp, (e) cerebellar and brainstem lesions or severe dysarthria, (f) severe audiovisual impairment, (g) other serious medical diseases or unstable conditions.

### 2.3. Outcome measures

In this study, WAB, as the primary outcome, was used to judge whether it was PSA and the severity. The AQ of WAB (WAB-AQ) was indirectly calculated from the scores of four sub-items of spontaneous, listening comprehension, repetition, and naming obtained through direct testing. For example, the evaluator provided scores of fluency and information content, which were added together to obtain the score of spontaneous speech, according to the scoring standards of WAB after the patient answered the questions. Combined with symptoms, medical history, and imaging data, stroke patients with a WAB-AQ lower than 93.8 points were judged as PSA, and the lower the WAB-AQ, the more severe the aphasia (Kertesz and Poole, 2004; Yan et al., 2022a). The secondary outcome measures included the Montreal Cognitive Assessment (MoCA), Fugl-Meyer Assessment (FMA), and Barthel Index (BI), which were selected to evaluate the cognitive function, motor function, and activities of daily living, respectively (Shah et al., 1989; Gladstone et al., 2002; Nasreddine et al., 2005). It should be pointed out that the evaluation of motor function was divided into FMA Upper Extremity (FMA-UE) and FMA Lower Extremity (FMA-LE) in this trial. The corresponding data were collected at baseline (T0) and week 3 (T1) by uniformly trained evaluators, who were blind.

### 2.4. Intervention

The random sequence was generated by the computer-generated random numbers. The random number was placed in a sealed and opaque envelope and implemented by the independent researcher. All subjects were divided into the conventional group (CG), MIT combined with sham stimulation group (SG), and MIT combined with tDCS group (TG) based on the random numbers.

CG: The speech therapist provided 30 min one-on-one training to the patient once a day, with a total dose of 15 sessions over 3 weeks, including voice induction, monosyllabic and polysyllabic word, and sentence pronunciation, picture naming, picture speaking, etc.

SG: (a) MIT: Each one-on-one training lasted for 30 min, with a total dose equivalent to conventional treatment. The patient was induced to use rhythmic hand tapping to facilitate the reproduction of items. MIT consisted of three levels, from words to phrases to sentences, with gradually increasing difficulty. Patients could not progress to the next difficulty level unless they could complete more than 95% five times in a row. The speech therapist gradually reduced assistance until the patient regained independent verbal expression in an almost normal manner. (b) Sham stimulation: The anode of tDCS acted on the left IFG of the cerebral hemisphere, and the cathode is placed on the right supra-brow frontal region. Each session only lasted for the first 30s and the duration was 15 sessions over 3 weeks.

TG: (a) MIT: same as SG; (b) tDCS: The location of electrodes was the same as SG. The stimulation intensity was 1 mA, lasting for 20 min per day, and the duration of tDCS was 15 sessions over 3 weeks.

The equipment used in this study was an ActivaDose®II portable transcranial direct current stimulator manufactured by ActivaTek Company of the United States.

### 2.5. Statistical analysis

Statistical analyses were conducted using SPSS 25.0 (IBM Corporation, Armonk, NY, United State). The mean values of three groups of continuous numerical variables were compared using one-way ANOVA. Categorical variables were expressed by rate, using Pearson’s Chi-square test. In the baseline characteristic analysis of this study, age, years of education, post-stroke duration, NIHSS, WAB, MoCA, FMA, and BI were statistically analyzed by one-way ANOVA, and gender was analyzed by Pearson’s Chi-square test. The paired sample T-test was used to statistically analyze the functional changes of each group of subjects before and after the intervention. The functional difference between the three groups of subjects before and after the intervention was statistically analyzed using the method of one-way ANOVA, and the variables with statistical differences among the three groups were further compared with each other using the *post hoc* test. A two-sided *p* < 0.05 was considered to be statistically significant in this study.

## 3. Results

### 3.1. Baseline characteristics

**Figure 1:**
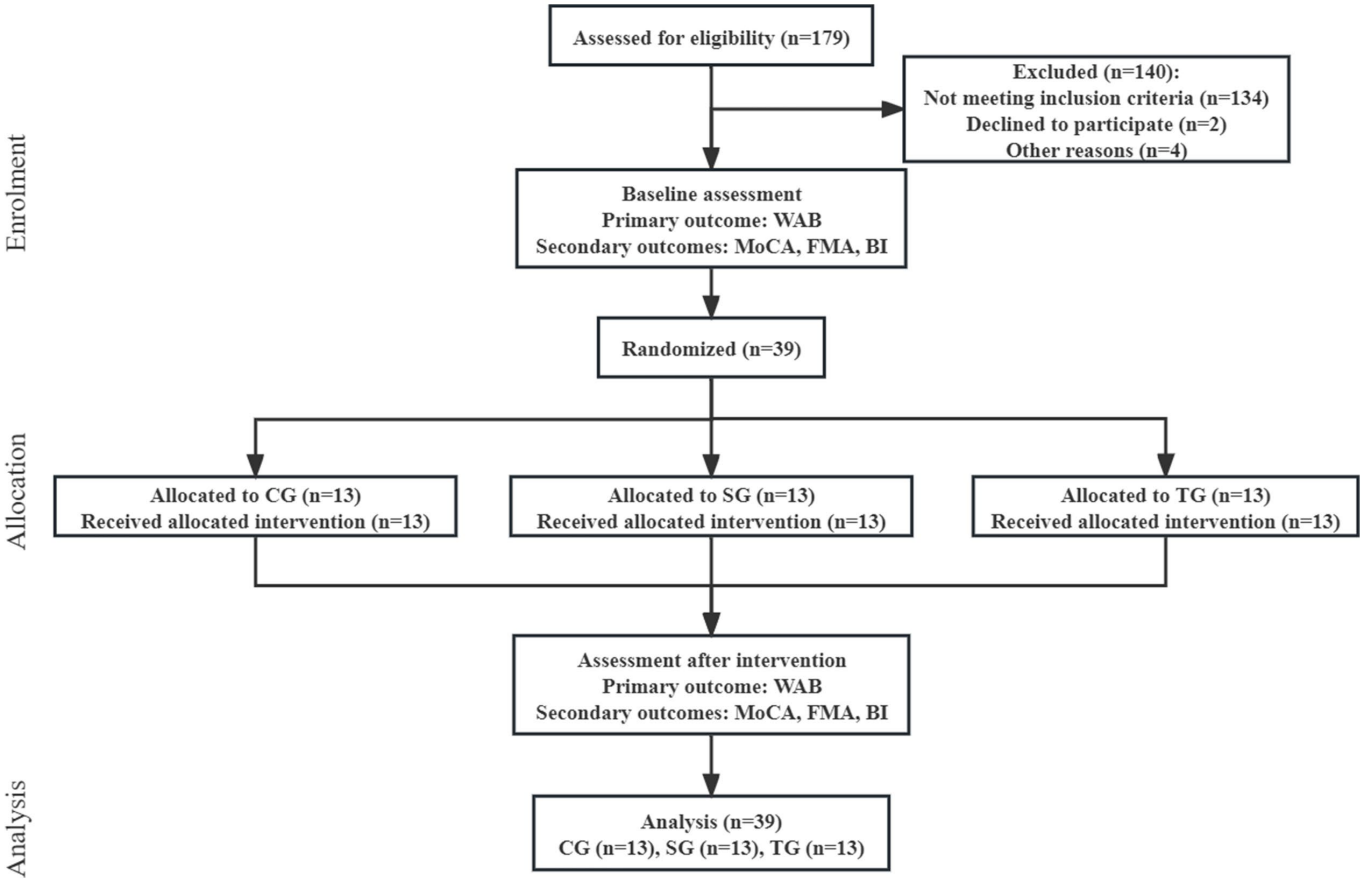
Flow diagram. WAB, Western Aphasia Battery; MoCA, Montreal Cognitive Assessment; FMA, Fugl-Meyer Assessment; BI, Barthel Index; CG, conventional group; SG, melodic intonation therapy combined with sham stimulation group; TG, melodic intonation therapy combined with transcranial direct current stimulation.

Between December 2021 and October 2022, we assessed 179 patients for eligibility. 39 patients (22%) were enrolled and randomly assigned to CG, SG, and TG according to the ratio of 1:1:1 (Figure 1). The participants had a mean (SD) age of 58.6 (11.1) years, had 11 (3) years of education, and were 5 (9) months from stroke onset. There were 17 (44%) females, and the median NIHSS score before intervention was 15 (IQR 13–19). The three arms before intervention had no statistical difference in demographic and clinical characteristics (Table 1).

**Table 1:** Baseline characteristics.

	CG (*n* = 13)	SG (*n* = 13)	TG (*n* = 13)	*p* value
Age (years)	56.77 ± 9.791	55.46 ± 12.204	63.46 ± 10.187	0.142^a^
Gender				0.174^b^
Female	6 (46.2)	3 (23.1)	8 (61.5)	
Male	7 (53.8)	10 (76.9)	5 (38.5)	
Education (years)	9.92 ± 2.842	11.54 ± 4.390	10.23 ± 2.488	0.434^a^
Post-stroke duration (days)	122.38 ± 299.172	92.23 ± 122.415	241.23 ± 373.781	0.381^a^
NIHSS	14.77 ± 7.585	15.54 ± 4.521	14.23 ± 5.510	0.857^a^
WAB-AQ	35.085 ± 37.906	30.138 ± 27.582	24.915 ± 26.579	0.709^a^
Spontaneous	6.38 ± 7.654	5.08 ± 6.006	3.38 ± 4.610	0.475^a^
Comprehension	85.31 ± 86.505	84.46 ± 63.763	100.08 ± 62.758	0.824^a^
Repetition	43.00 ± 45.200	37.85 ± 39.179	22.85 ± 38.773	0.439^a^
Naming	25.92 ± 40.323	16.77 ± 24.749	17.85 ± 30.506	0.737^a^
MoCA	4.62 ± 6.935	3.32 ± 6.379	3.62 ± 5.268	0.844^a^
FMA	31.15 ± 24.283	20.62 ± 20.974	31.31 ± 24.295	0.414^a^
FMA-UE	17.00 ± 15.875	9.77 ± 14.143	15.69 ± 15.628	0.444^a^
FMA-LE	14.15 ± 9.063	10.85 ± 8.153	15.62 ± 11.012	0.431^a^
BI	38.46 ± 23.838	33.46 ± 17.957	46.92 ± 28.250	0.354^a^

Data are expressed as mean ± SD or number of observations (% of total observation). NIHSS, National Institute of Health Stroke Scale, WAB-AQ, aphasia quotient of Western Aphasia Battery; MoCA, Montreal Cognitive Assessment; FMA, Fugl-Meyer Assessment; FMA-UE, Fugl-Meyer Assessment Upper Extremity; FMA-LE, Fugl-Meyer Assessment Lower Extremity; BI, Barthel Index; CG, conventional group; SG, melodic intonation therapy combined with sham stimulation group; TG, melodic intonation therapy combined with transcranial direct current stimulation.

a 1-way analysis of variance.

b Pearson’s Chi-square test.

### 3.2. Comparison of functional values before and after intervention in each group

**Figure 2:**
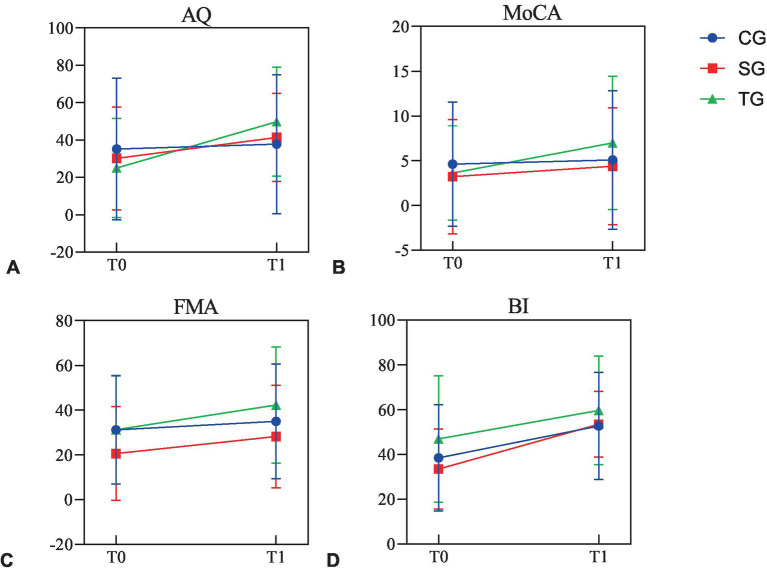
The functional values before and after intervention in three groups. **(A)** The values of AQ before and after intervention in three groups. **(B)** The values of MoCA before and after intervention in three groups. **(C)** The values of FMA before and after intervention in three groups. **(D)** The values of BI before and after intervention in three groups. AQ, aphasia quotient; MoCA, Montreal Cognitive Assessment; FMA, Fugl-Meyer Assessment; BI, Barthel Index; CG, conventional group; SG, melodic intonation therapy combined with sham stimulation group; TG, melodic intonation therapy combined with transcranial direct current stimulation; T0, baseline; T1, week 3.

In CG, except for WAB-AQ (37.746 ± 37.172, *p* = 0.06) and MoCA (5.08 ± 7.740, *p* = 0.190), FMA (35.00 ± 25.668, *p* = 0.008) and BI (52.69 ± 23.859, *p* < 0.001) were improved after intervention, which was statistically significant. The analysis results of sub-items in WAB and FMA showed that there was no statistical difference in other sub-items except listening comprehension (89.62 ± 86.358, *p* = 0.017), FMA-UE (19.38 ± 16.556, *p* = 0.019), and FMA-LE (15.62 ± 9.760, *p* = 0.017). In SG and TG, the functional value changes of WAB-AQ, MoCA, FMA, and BI before and after intervention were statistically significant, including the sub-items of spontaneous, listening comprehension, repetition, naming, FMA-UE, and FMA-LE (*p* < 0.05; Table 2; Figure 2).

**Table 2:** Comparison of functional values before and after intervention in each group.

	CG (*n* = 13)		*p* value	SG (*n* = 13)		*p* value	TG (*n* = 13)		*p* value
	T0	T1		T0	T1		T0	T1	
WAB-AQ	35.085 ± 37.906	37.746 ± 37.172	0.060^a^	30.138 ± 27.582	41.323 ± 23.526	0.004^a^	24.915 ± 26.579	49.715 ± 29.183	<0.001^a^
Spontaneous	6.38 ± 7.654	7.23 ± 7.339	0.059^a^	5.08 ± 6.006	8.15 ± 4.705	0.005^a^	3.38 ± 4.610	8.62 ± 5.938	<0.001^a^
Comprehension	85.31 ± 86.505	89.62 ± 86.358	0.017^a^	84.46 ± 63.763	99.85 ± 52.451	0.019^a^	100.08 ± 62.758	139.08 ± 50.457	0.001^a^
Repetition	43.00 ± 45.200	44.23 ± 43.43.924	0.400^a^	37.85 ± 39.179	53.00 ± 38.240	0.015^a^	22.85 ± 38.773	54.00 ± 41.002	0.004^a^
Naming	25.92 ± 40.323	27.38 ± 40.582	0.288^a^	16.77 ± 24.749	22.15 ± 25.848	0.032^a^	17.85 ± 30.506	39.54 ± 34.374	0.009^a^
MoCA	4.62 ± 6.936	5.08 ± 7.740	0.190^a^	3.23 ± 6.379	4.38 ± 6.526	0.007^a^	3.62 ± 5.268	7.00 ± 7.439	0.016^a^
FMA	31.15 ± 24.283	35.00 ± 25.668	0.008^a^	20.62 ± 20.974	28.23 ± 22.873	0.004^a^	31.31 ± 24.295	42.31 ± 26.033	<0.001^a^
FMA-UE	17.00 ± 15.875	19.38 ± 16.556	0.019^a^	9.77 ± 14.143	15.23 ± 15.595	0.008^a^	15.69 ± 15.628	23.92 ± 18.301	0.001^a^
FMA-LE	14.15 ± 9.063	15.62 ± 9.760	0.017^a^	10.85 ± 8.153	13.00 ± 8.287	0.004^a^	15.62 ± 11.012	18.38 ± 9.483	0.011^a^
BI	38.46 ± 23.838	52.69 ± 23.859	<0.001^a^	33.46 ± 17.957	53.46 ± 14.632	<0.001^a^	46.92 ± 28.250	59.62 ± 24.278	<0.001^a^

Data are expressed as mean ± SD. WAB-AQ, aphasia quotient of Western Aphasia Battery; MoCA, Montreal Cognitive Assessment; FMA, Fugl-Meyer Assessment; FMA-UE, Fugl-Meyer Assessment Upper Extremity; FMA-LE, Fugl-Meyer Assessment Lower Extremity; BI, Barthel Index; CG, conventional group; SG, melodic intonation therapy combined with sham stimulation group; TG, melodic intonation therapy combined with transcranial direct current stimulation; T0, baseline; T1, week 3.

a Paired sample *T*-test.

### 3.3. Comparison of functional differences among three groups

The difference of WAB-AQ (*p* < 0.001), MoCA (*p* = 0.024), and FMA (*p* = 0.044) before and after the intervention was statistically different among the three groups, but BI (*p* = 0.154) was not. The statistical analysis of sub-items in WAB and FMA showed that there were statistical differences in the functional differences among the three groups in spontaneous (*p* = 0.003), listening comprehension (*p* = 0.001), repetition (*p* = 0.005), naming (*p* = 0.005), and FMA-UE (*p* = 0.041), but FMA-LE (*p* = 0.432) was not (Table 3).

**Table 3:** Comparison of functional differences among three groups.

	CG (*n* = 13)	SG (*n* = 13)	TG (*n* = 13)	*p* value
ΔWAB-AQ	2.662 ± 4.624	11.185 ± 11.304	24.800 ± 18.420	<0.001^a^
ΔSpontaneous	0.85 ± 1.463	3.08 ± 3.278	5.23 ± 3.767	0.003^a^
ΔComprehension	4.31 ± 5.603	15.38 ± 20.394	39.00 ± 31.815	0.001^a^
ΔRepetition	1.23 ± 5.085	15.15 ± 19.200	31.15 ± 31.704	0.005^a^
ΔNaming	1.46 ± 4.737	5.38 ± 7.995	21.69 ± 25.131	0.005^a^
ΔMoCA	0.46 ± 1.198	1.15 ± 1.281	3.38 ± 4.331	0.024^a^
ΔFMA	3.85 ± 4.356	7.62 ± 7.859	11.00 ± 8.073	0.044^a^
ΔFMA-UE	2.38 ± 3.176	5.46 ± 6.200	8.23 ± 6.870	0.041^a^
ΔFMA-LE	1.46 ± 1.898	2.15 ± 2.193	2.77 ± 3.320	0.432^a^
ΔBI	14.23 ± 10.576	20.00 ± 10.408	12.69 ± 8.567	0.154^a^

Data are expressed as mean ± SD. Δ, subtract the baseline score from the 3-week score; WAB-AQ, aphasia quotient of Western Aphasia Battery; MoCA, Montreal Cognitive Assessment; FMA, Fugl-Meyer Assessment; FMA-UE, Fugl-Meyer Assessment Upper Extremity; FMA-LE, Fugl-Meyer Assessment Lower Extremity; BI, Barthel Index; CG, conventional group; SG, melodic intonation therapy combined with sham stimulation group; TG, melodic intonation therapy combined with transcranial direct current stimulation.

a 1-way analysis of variance.

**Figure 3:**
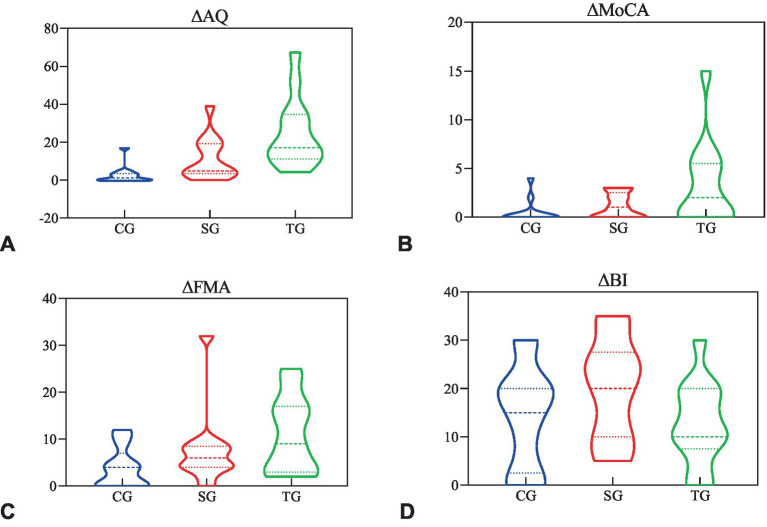
The functional difference before and after the intervention. **(A)** The difference of AQ before and after intervention in three groups. **(B)** The difference of MoCA before and after intervention in three groups. **(C)** The difference of FMA before and after intervention in three groups. **(D)** The difference of BI before and after intervention in three groups. Δ, subtract the baseline score from the 3-week score; AQ, aphasia quotient; MoCA, Montreal Cognitive Assessment; FMA, Fugl-Meyer Assessment; BI, Barthel Index; CG, conventional group; SG, melodic intonation therapy combined with sham stimulation group; TG, melodic intonation therapy combined with transcranial direct current stimulation.

The *post hoc* test results of ANOVA showed that the changes of WAB-AQ and MoCA in TG before and after the intervention were more significant than CG (*p* < 0.001, *p* = 0.009) and SG (*p* = 0.01, *p* = 0.042), and the change of FMA in TG was more significant than CG (*p* = 0.013), but there was no statistical difference between TG and SG (*p* = 0.224; Figure 3). In terms of spontaneous and repetition, there was a significant difference between TG and CG (*p* = 0.001, *p* = 0.001), while there was no significant difference between TG and SG (*p* = 0.076, *p* = 0.067). In terms of comprehension and naming, there were significant differences between TG and CG (*p* < 0.001, *p* = 0.002) and SG (*p* = 0.01, *p* = 0.011). As for FMA-UE, TG showed a more significant improvement than TG (*p* = 0.012), while there were no statistical differences between the other groups.

No serious adverse events occurred during the whole study.

## 4. Discussion

In this trial, tDCS, as a central stimulus, activated the left dominant hemisphere language functional area through anodic stimulation, and MIT, as a peripheral stimulus, strengthened positive feedback through continuous input of signals including motion, vision, and hearing. Together, they formed the complete closed-loop treatment system for aphasia to explore the clinical efficiency of the closed-loop scheme on functional recovery and provide a clinical reference for linguistic rehabilitation. The results of this study revealed that, immediately after the intervention, both SG and TG improved in terms of language function, while CG was not significantly. Although SG and TG had a similar effect on spontaneous and repetition, SG was inferior to TG in comprehension and naming, and the overall efficiency of TG was better than SG.

Language recovery was related to nervous system reorganization (Stefaniak et al., 2020). The review showed that the closed-loop rehabilitation system could augment the positive effect of simple peripheral or central stimulation (Jia, 2022), which was related to a previous study that concluded that peripheral intervention combined with central intervention enhanced synaptic plasticity more strongly than any individual intervention (Fritsch et al., 2010). Based on extensive activation of the cerebral cortex by tDCS, TG, combined with behavioral training, enhanced the plasticity of synapses participating in specific tasks, which would appear in the short term and continue in the long term (Fridriksson and Hillis, 2021). This result provided an effective evidence basis for the clinical application of closed-loop rehabilitation of aphasia. Spontaneous neuroplasticity, which could be affected by therapy, was the norm in the early poststroke period (Dabrowski et al., 2019). Remarkably, the mean post-stroke duration of TG is about 8 months, which was longer than the other two groups in this trial, but the therapeutic efficacy of TG was stronger. It might be another piece of evidence supporting closed-loop rehabilitation. In the future, it is necessary to continue to carry out stratified research according to the course of the disease. Additionally, although SG was superior to CG, it had limitations in integrally improving communication. 112.5 h of MIT intervention promoted language output but not comprehension (Marchina et al., 2023). Moreover, due to previous studies focusing on behavioral indicators and insufficient imaging evidence, the neurological effect of MIT on aphasia patients was still unclear (Zhang X. Y. et al., 2021; Zhang X. et al., 2021). In this study, there was also a lack of neuroimaging detection, and the potential neural recovery mechanism of closed-loop rehabilitation also needed to be further supplemented.

The results of cognitive function analysis in this study showed that both SG and TG significantly improved after intervention, but CG was not. Moreover, the *post hoc* test supported the result that TG had the most significant effect on promoting cognitive function. The above results had a similar development tendency with the analysis of language function. Cognition is the general term for the process of recognizing and understanding objects, which belongs to the high-level activities of the cerebral cortex and interacts with language function (Norris et al., 2016; Ardila and Rubio-Bruno, 2018). Some studies had shown that the cognitive level of patients with aphasia after stroke was lower than that of non-aphasic patients, and there was a positive correlation between cognition and language (Fonseca et al., 2019; Yan et al., 2022b). These explained the experimental results and provided evidence for cognition-language integration intervention, which might be further explored in future research.

In language processing, language-related gestures might promote language performance (Grechuta et al., 2019). In our study, we found that although motor function improved in all three groups after treatment, SG and TG showed differences from CG in FMA and FMA-UE. In addition, a positive correlation between language and upper limb motor function was also found in the cross-sectional study (Xu et al., 2021). These suggested that the behavioral therapy paradigm in this trial might also facilitate motor function recovery, especially in the upper limbs, which needed to be verified by larger studies in the future.

The reason why the difference of BI was not statistically different among the three groups might be the lack of language components in BI, and the closed-loop rehabilitation program designed in this study mainly aimed at promoting the recovery of language function. In addition, the ability of daily living reflected by BI could be affected by other non-language factors, such as motor function, auxiliary appliance application, functional compensation, etc.

## 5. Conclusion

MIT combined with tDCS or sham stimulation is effective for patients with PSA, but the conventional treatment cannot significantly improve aphasia in the short term. Compared with sham stimulation, the closed-loop rehabilitation scheme combining MIT and tDCS can augment the positive effect on language and cognitive recovery in PSA and deserves promotion in clinical practice.

## Data availability statement

The original contributions presented in the study are included in the article/supplementary material, further inquiries can be directed to the corresponding authors.

## Ethics statement

The studies involving human participants were reviewed and approved by the Ethics Committee of the Third Affiliated Hospital of Xinxiang Medical University. The patients/participants provided their written informed consent to participate in this study.

## Author contributions

ZY and JJ conceived and designed the analysis and writing – original draft. ZY, XH, MC, XF, and DW collected the data. ZY, XH, MC, XF, DW, SX, CL, XL, HX, and JJ contributed data or analysis tools, writing – review and editing, and discussed the results. ZY and XH performed the analysis. All authors contributed to the article and approved the submitted version.

## Funding

This work was supported by the National Key R&D Program of China (2018YFC2002300), the National Natural Innovation Research Group Project of China (82021002), and the National Natural Major Research Program Integration Project of China (91948302).

## Conflict of interest

The authors declare that the research was conducted in the absence of any commercial or financial relationships that could be construed as a potential conflict of interest.

## Publisher’s note

All claims expressed in this article are solely those of the authors and do not necessarily represent those of their affiliated organizations, or those of the publisher, the editors and the reviewers. Any product that may be evaluated in this article, or claim that may be made by its manufacturer, is not guaranteed or endorsed by the publisher.
